# Dissociation of Brain Activation in Autism and Schizotypal Personality Disorder During Social Judgments

**DOI:** 10.1093/schbul/sbx083

**Published:** 2017-06-21

**Authors:** Andrew C Stanfield, Ruth C M Philip, Heather Whalley, Liana Romaniuk, Jeremy Hall, Eve C Johnstone, Stephen M Lawrie

**Affiliations:** 1 Patrick Wild Centre, University of Edinburgh, Edinburgh, UK; 2 Division of Psychiatry, University of Edinburgh, Edinburgh, UK; 3 Tailor Ed Foundation, Edinburgh, UK; 4 Institute of Psychological Medicine and Clinical Neurosciences, University of Cardiff, Cardiff, UK

**Keywords:** social cognition, negative symptoms, imaging/fMRI

## Abstract

**Background:**

There are overlaps between autism and schizophrenia but these are particularly pronounced, especially in social domains, for higher functioning individuals with autism spectrum disorders (ASD) or schizotypal personality disorder (SPD). It is not known whether these overlapping social deficits result from shared or distinct brain mechanisms. We therefore compared social cognition in ASD and SPD using functional magnetic resonance imaging (fMRI).

**Methods:**

Twenty-one individuals with SPD, 28 with ASD and 33 controls were compared with respect to clinical symptoms using the Positive and Negative Syndrome Scale; social cognition, using a social judgment task and Ekman 60 faces task; and brain activation using an fMRI task of social judgment.

**Results:**

The ASD and SPD groups showed few differences in symptoms or social cognition. However, fMRI showed that, compared to ASD, the SPD group showed significantly greater activation during social compared to gender judgments in the amygdala and 3 clusters: right posterior cerebellum, extending into fusiform and inferior temporal gyri; left posterior cerebellum; and left intraparietal sulcus extending through medial portions of the temporal gyri into the fusiform gyrus (all *P* < .05 family-wise error corrected). Control activations lay between the ASD and SPD groups.

**Conclusions:**

Although social cognitive deficits in ASD and SPD appear superficially similar they are the result of different brain mechanisms. These findings have implications for therapeutic interventions targeted at social dysfunction in these conditions.

## Introduction

The term autism was initially coined by Bleuler in 1911 to describe a characteristic symptom of people with schizophrenia, specifically “detachment from reality, together with the relative and absolute predominance of the inner life.”^1^ It was first used to describe a specific disorder by Kanner in 1943, when he presented a case series of children affected by “autistic disturbance of affective contact.”^2^ Although initially thought to be a distinct condition, autism soon came to be regarded as a form of early onset schizophrenia^3^ and this continued until a series of studies differentiated the disorders on phenomenology, course and family history.^4–9^

With the advent of the autism spectrum concept, it is now recognized that there exist forms of both disorders which do not show such marked impairments. Although autism spectrum disorders (ASD) and “schizophrenia spectrum disorders,” such as schizotypal personality disorder (SPD), would be expected to differ on the level of mild psychotic symptoms and on restricted repetitive behaviors,^10^ there are significant overlaps between the conditions: both occur in nonintellectually disabled people and are associated with social difficulties, idiosyncratic language and unusual behavior, as well as showing common associated psychopathology.^11–24^ Both conditions are also associated with deficits in social cognition.^25–31^ Finally, the age of onset of SPD is unclear, while ASD may not become obvious until after early childhood, when social demands exceed ability.^10^ Thus the distinction between ASD and SPD can be difficult^32–34^; indeed it has been proposed that the disorders should not be classified separately.^35^

Clinical and neuropsychological similarities therefore exist between ASD and SPD, but it is unclear whether these share a common pathophysiological mechanism, as direct comparisons have not been conducted. It has been suggested that, although ASD and schizophrenia show similar social deficits, the mechanisms through which these develop differ, with schizophrenia associated with hyper-mentalizing (ie, over-ascription of mental states to others) and ASD associated with hypo-mentalizing.^36–40^ To the authors’ knowledge, 3 functional magnetic resonance imaging (fMRI) studies have directly compared ASD and schizophrenia using social cognition tasks^41–43^; these are broadly supportive of the hypo-/hyper-mentalizing theory, particularly the most recent studies.^41,44^ However, it is also not clear whether these findings apply to higher functioning groups with ASD and SPD, in which fewer symptomatic differences are apparent.

We therefore compared social cognitive deficits in people with ASD and SPD and tested whether they are associated with different underlying brain activity using fMRI. We employed a social judgment task (assessing approachability from faces) on which we have previously shown impaired performance in ASD^27^ and schizophrenia.^45^ Making a judgment of approachability requires individuals to assess affective information from facial cues and to interpret this in relation to the threat or otherwise represented.^46^ Using fMRI, we have also shown this task to activate social brain regions in typically developing individuals, including the medial and inferior prefrontal cortex, amygdala and cerebellum.^46^ We hypothesized that individuals with ASD and those with SPD would show impaired social judgment compared to controls, but that, consistent with the literature on autism and schizophrenia, those with SPD would show increased activation of these brain regions while making social judgments whereas the opposite pattern would be seen in ASD.

## Methods

### Participants

Individuals with ASD were recruited from clinical and support services in Southeast Scotland. All had a DSM-IV diagnosis of either autism or Asperger Syndrome and met ASD cut-offs on the Autism Diagnostic Observational Schedule (ADOS-G).^47^

Participants with SPD were recruited from nonpsychotic people who had previously participated in the Edinburgh High Risk Study of schizophrenia (EHRS)^48^ and from clinical services in Southeast Scotland. All met DSM-IV criteria for SPD using the Structured Clinical Interview for DSM-IV Axis II Disorders (SCID-II).^49^

Some individuals met criteria for both ASD (determined by DSM-IV and the ADOS) and SPD (determined by the SCID-II). These were analyzed as a separate group, referred to as “comorbid” (CM).

Controls were recruited from participant and investigator acquaintances and the Scottish Mental Health Network research register. Individuals with a history of, or first degree relative with, ASD, SPD or a psychotic illness were excluded.

General exclusion criteria were IQ < 70, substance dependence or history of schizophreniform disorder, schizophrenia or bipolar affective disorder.

The study was approved by the NHS Lothian Research Ethics Committee. Written informed consent was obtained from all participants.

### Assessments

In addition to the ADOS-G and the SCID-II, participants were assessed using the Positive and Negative Syndrome Scale (PANSS)^50^ and the Wechsler Abbreviated Intelligence Scale.^51^ For those on antipsychotic medication, doses were converted to chlorpromazine equivalents.^52,53^

Social cognition was assessed outside the MRI scanner using the Ekman 60 facial emotion recognition test^54^ and a social judgments task.^45^ In the Ekman 60 each face was presented for up to 5 seconds and participants selected the emotion displayed from a randomly ordered list consisting of fear, anger, disgust, sadness, happiness and surprise. Ten presentations of each emotion were shown in a random order. Performance was measured by totaling correctly identified emotion labels.

For the social judgment task, participants were shown 6 sets of 32 faces for up to 5 seconds each. In each set they allocated the faces into one of the following binary characteristics: approachable-unapproachable, distinctive-not distinctive, young-old, trustworthy-untrustworthy, intelligent-not intelligent, and attractive-unattractive. The stimuli for the social judgment task were the same as a previous study and ratings for each were scored as “correct” when they agreed with predefined ratings for each stimulus.^45^

### fMRI Image Acquisition

Details of image acquisition and preprocessing are given in the supplementary material.

### fMRI Approachability Task

The approachability component of the social judgment task was adapted for the scanner as previously described.^46^ Face stimuli were presented in blocks of approachability judgments (“social” condition) and gender judgments (“gender” condition). Stimuli differed from those employed for the behavioral task. Two runs were presented, each lasting 240 seconds. Three blocks of each condition were shown; each lasted for 25 seconds, separated by a central fixation cross (“Baseline” condition). Each block began with a 1 second visual reminder of the task for the block (“Approachable?” or “Gender?”), followed by 6 faces, in a pseudorandom order, each presented for 3.5 seconds with a 0.5-second gap between stimuli. Underneath the faces, participants were shown their bivalent choice (“Approachable:Not approachable” or “Male:Female”) and indicated their selection by pressing a button in the hand corresponding to their choice. The stimuli were counterbalanced for stimulus order, judgment order, and hand used to indicate choice.

### Data Analysis

Differences between demographic characteristics were determined using parametric or nonparametric tests. The PANSS, Ekman 60, and social judgment scores were not normally distributed and so were analyzed using Kruskal-Wallis tests. When significant results were identified in the Kruskal-Wallis tests, follow-up Mann Whitney *U* tests were conducted. To assess the potential confounding effect of IQ, partial correlations between IQ and performance were conducted across all participants with group as a covariate.

Statistical analysis of fMRI data were conducted using the general linear model in SPM8. Data for individual participants were modeled with 3 conditions (social judgment, gender judgment and baseline). Parameters representing participant movement were entered as covariates of no interest. Contrast images were generated for each participant for 2 contrasts: social vs baseline and gender vs baseline. In the second level analysis, a 2 × 4 flexible factorial design matrix was constructed with the 2 contrasts (social vs baseline and gender vs baseline) as within subjects factors, and 4 groups (ASD, SPD, CM, and control) as between-subjects factors, in addition to subject constants. Contrasts were constructed to test the main effect of condition (social or gender) across all 4 groups; the effect of condition within each group; and the group × condition interaction. Note that the group × interaction contrast essentially allows comparison of the social and gender conditions, with the gender condition acting as a “high level” baseline to remove the effects of any differential face processing not related to affective content.

Between group analyses were conducted using an initial height threshold of *P* = .005 uncorrected. Cluster results were only considered significant at *P* < .05 after family wise error (FWE) correction for multiple comparisons across the whole brain. A small volume correction (SVC) was applied to the amygdala bilaterally.

When clusters showed a significant group × condition interaction, eigenvariates were extracted and the difference value calculated by subtracting the value for the gender vs baseline contrast from the social vs baseline contrast. These difference values were regressed against PANSS scores to explore the relationship between brain activation and symptomatology. To assess the effect of potential confounding factors, difference values were regressed against IQ, chlorpromazine equivalents and task performance. Regression analyses were conducted within IBM SPSS Statistics 19.0. Finally, to examine whether results related to differences in activation during the social or nonsocial condition, or both, eigenvariates for the social vs baseline and gender vs baseline conditions were compared between groups.

## Results

### Participants

Characteristics of the participants are given in table 1.

**Table 1.** Participant Characteristics

	ASD	SPD	CM	Controls
*N*	28	21	10	33
M:F	22:6	14:7	7:3	23:10
Age	39.5 (11.6)	37.1 (9.2)	34.9 (9.9)	36.5 (9.3)
Handedness	27:1	19:2	8:2	31:2
Years education	16.2 (1.7)	15.2 (2.0)	16.2 (2.3)	16.5 (1.9)
Full-scale IQ*	113.1 (17.3)	106.4 (10.7)	103.5 (22.5)	118.1 (9.9)
Antipsychotic use (yes:no)*	2:26	5:16	3:7	0:33

*Note*: ASD, autism spectrum disorder; SPD, schizotypal personality disorder; CM, comorbid.

*Differed significantly between groups (*P* < .05).

No significant differences were seen with respect to gender, handedness, age or education (all *P* > .22). IQ scores differed significantly (*F* = 4.12, *P* = .009) with the control group having significantly higher IQ than the SPD and the CM group (all *P* < .05). The ASD, SPD and CM groups did not differ significantly on IQ (all *P* > .08). Ten participants were taking antipsychotic medication in chlorpromazine equivalent doses ranging from 25 mg to 400 mg per day. The median chlorpromazine equivalent doses for those taking antipsychotics in each group were: ASD = 50 mg, SPD = 100 mg, CM = 150 mg. The SPD and the CM groups were more likely to be taking antipsychotic medication than the ASD or control groups (*P* = .008).

### Clinical Features

**Fig. 1.**
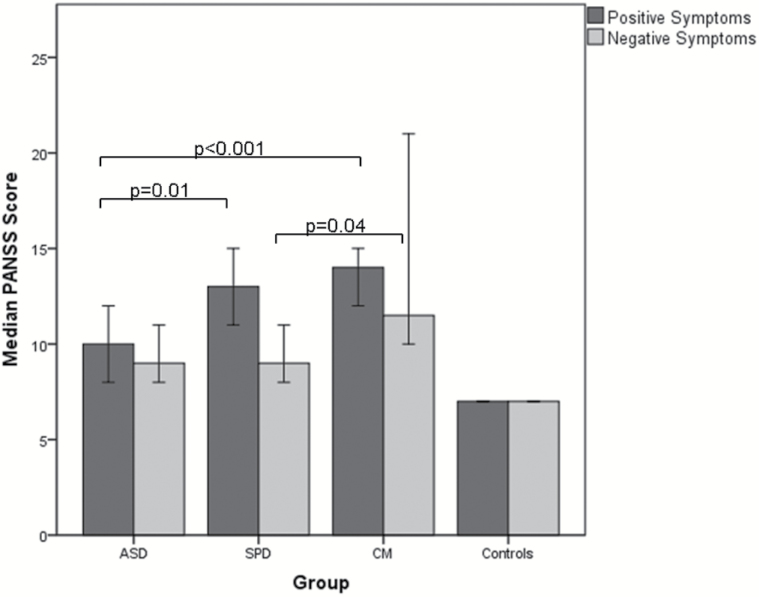
Median Positive and Negative Syndrome Scale (PANSS) positive and negative symptom scores. Error bars represent 95% CIs.

Summary scores for PANSS positive and negative symptom scales are shown in figure 1.

Kruskal-Wallis tests showed significant differences between the groups for positive and negative symptoms (χ^2^ = 49.3, *P* < .001 and χ^2^ = 41.7, *P* < .001, respectively). Follow-up Mann-Whitney tests showed that the ASD group scored less than the SPD and CM groups on positive symptoms (*Z* = −3.34, *P* = .01; *Z* = −3.7, *P* < .001, respectively). With respect to negative symptoms, there was no difference between the ASD and SPD group (*Z* = −.82, *P* = .41); however, the CM group scored significantly more than the SPD group (*Z* = −2.0, *P* = .04) and showed a trend towards a significantly higher score than the ASD group (*Z* = −1.7, *P* = .09).

### Social Cognition

The results for the out of scanner social cognition tasks are summarized in supplementary table s1.

In the Ekman 60, there were no significant differences between the ASD, SPD, and CM groups on any measure. The ASD group identified significantly fewer angry faces correctly than the controls (*P* = .002), while the ASD, SPD, and CM groups all identified significantly fewer fearful faces correctly than the controls (all *P* < .05). A significant positive relationship was seen across the groups between IQ and anger recognition (*P* < .001) suggesting differences in this measure may relate to IQ differences between the groups; no such relationship was seen for fear.

In the Social Judgments Task, the ASD, SPD, and CM groups did not differ significantly from each other on any of the measures. The ASD and SPD groups both scored significantly less than the controls on judgments of approachability, attractiveness, distinctiveness and intelligence (all *P* < .05). The CM group scored significantly less than controls on judgments of age and distinctiveness (*P* < .02 for both). IQ correlated positively with scores on age and distinctiveness (*P* = .01 and *P* = .03, respectively), suggesting differences in these measures may relate to IQ differences between the groups.

### Functional Magnetic Resonance Imaging

Two individuals from the ASD group, 1 from the SPD group and 1 from the CM group did not participate in the imaging component due to fear of the scanner environment. Two individuals (1 control, 1 ASD) were excluded due to technical issues such that meaningful data were not recorded. Finally, one individual with ASD was excluded due to imaging artifacts. Supplementary table s2 contains the details of those included in the scanning study.

#### Task Performance and Within Group Analyses.

Details of in-scanner performance in the task and the within group analyses are in the supplementary material (supplementary tables s3–s6 and figures s1–s5). Within the whole study group combined, greater activations were found in the social compared to the gender condition in many regions previously associated with social brain function: inferior frontal gyri, medial prefrontal cortex, left anterior temporal lobe, left superior temporal sulcus, occipital gyri, and the cerebellum. No regions showed greater activation in the gender vs the social condition.

#### ASD, SPD, CM vs Controls.

There were no significant group × condition interactions in the ASD, SPD or CM vs control comparisons. However, in the ASD vs control comparison, 2 trends towards significant group × condition interactions were observed, with the ASD group showing less increase in activation than the controls during the social condition compared to the gender condition in the posterior cerebellum bilaterally (cluster peaks (30 −58 −44), *P* = .05; and (−45 −55 −41), *P* = .07; table s7 and figure s6 in supplementary material).

#### ASD vs SPD.

**Fig. 2.**
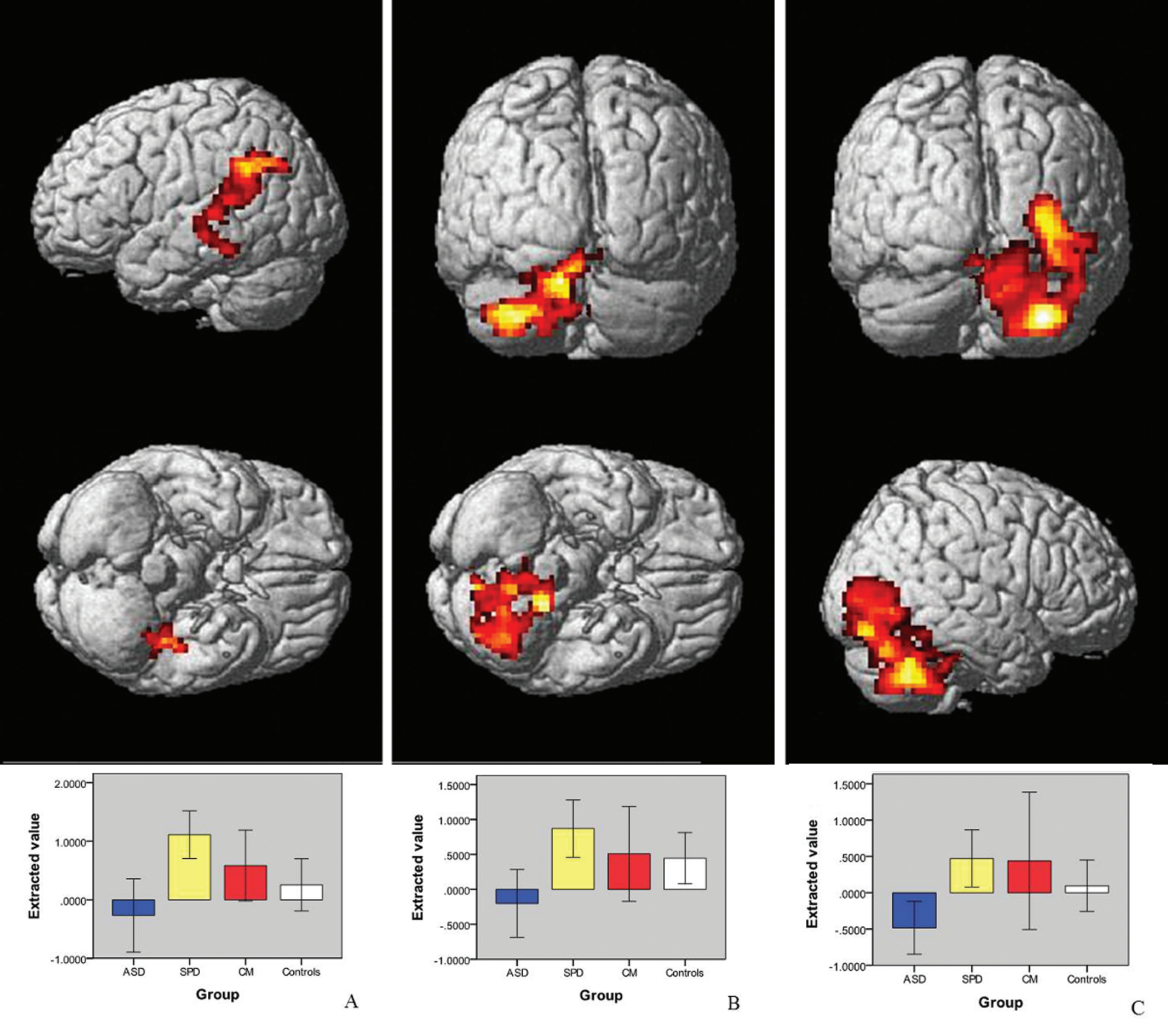
Clusters projected onto a rendered brain demonstrating regions of greater increase in activation in schizotypal personality disorder (SPD) compared to the autism spectrum disorder (ASD) group using the social > gender contrast in: (A) left temporo-parietal cluster (−24 −52 31); (B) left cerebellum (−15 −40 −38); (C) right cerebellum (33 −64 −44). All clusters were significant at an initial height threshold of *P* < .005 uncorrected with a cluster significance of *P* < .05 family wise error (FWE) corrected. Graphs underneath show difference values of extracted eigenvariates for each cluster.

**Fig. 3.**
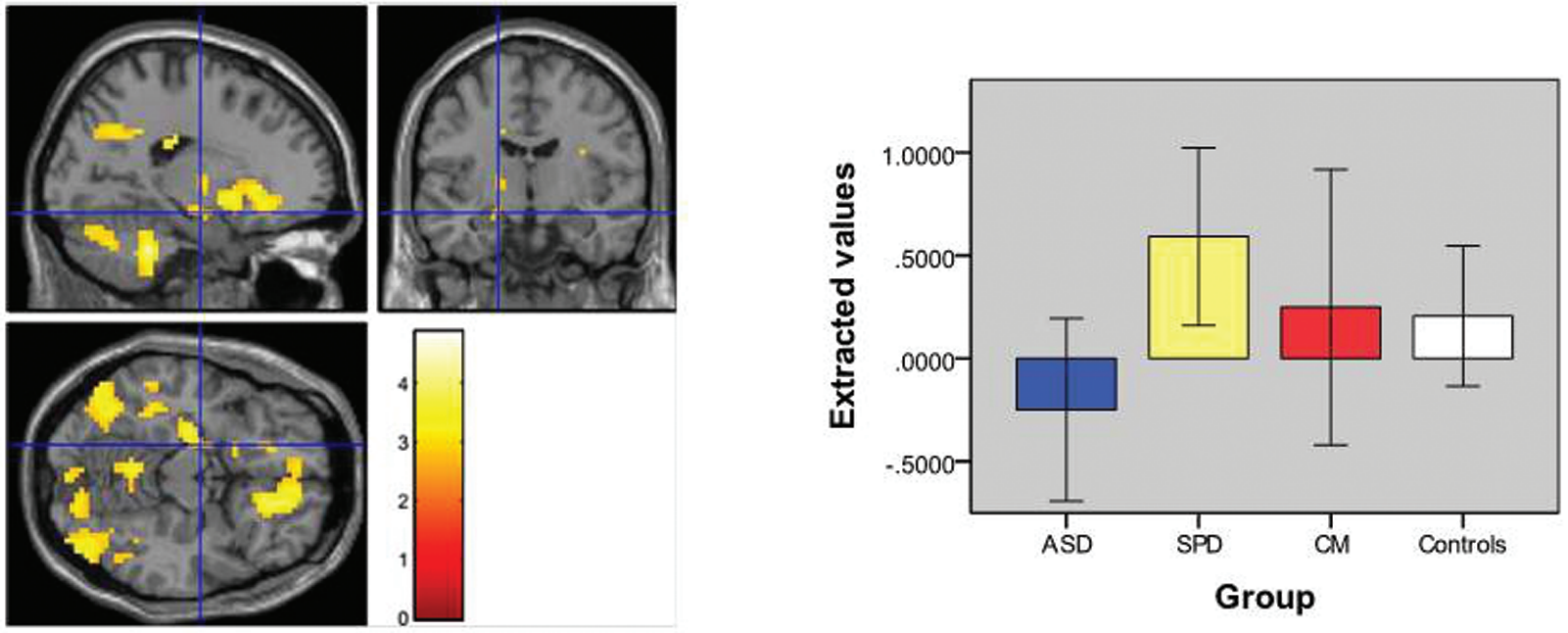
Location of peak voxel (*P* = .03 family wise error [FWE] corrected) of increased amygdala activation (−18 10 14) and graph of difference values of extracted eigenvariates for social > gender contrast in schizotypal personality disorder (SPD) vs autism spectrum disorder (ASD).

A significant group × condition interaction was seen for the ASD vs SPD comparison. The SPD group showed significantly greater activation compared to the ASD group when making social compared to gender judgments in a voxel in the amygdala and in 3 clusters: the right posterior cerebellum, extending into the fusiform and inferior temporal gyri; the left posterior cerebellum; and the left intraparietal sulcus extending through the medial portions of the temporal gyri into the fusiform gyrus. For each of these regions the controls lay between the ASD and the SPD groups (figures 2 and 3; table s8 and figure s7 in the supplementary material).

Due to recent concerns expressed about the possibility of false positives due to the use of cluster-based statistics in resting state fMRI,^55^ we also examined data for this comparison using voxel-based inference with a height threshold of *P* < .05 FWE corrected, which has not been found to show the same concerns.^55^ In this case, in addition to the significant voxel in the amygdala, we identified significant voxels in the right cerebellum at the same location as in our main analysis (*Z* = 4.54, *P* = .03) and in the right inferior frontal gyrus (MNI = 51 35 25, *Z* = 4.5, *P* = .03).

#### ASD vs CM.

A significant group × condition interaction was observed in the ASD vs CM contrast. During the social condition compared to the gender condition the CM group showed significantly greater increases in activation than the ASD group in left pre- and post-central gyri and right cerebellum (supplementary table s9).

#### SPD vs CM.

There were no significant group × condition interactions for the SPD vs control contrast.

#### Analysis of Confounding Factors.

No significant relationships were seen between fMRI activations and IQ, antipsychotic use or within-scanner task performance suggesting that results are not confounded by these factors. To further explore the effects of antipsychotic medication on the fMRI results, the ASD vs SPD analysis was repeated after omitting those taking antipsychotic medication. In this analysis, greater activation was seen in the SPD then in the ASD group in the cerebellum bilaterally and in a new cluster in the ventromedial prefrontal cortex (tables s10–s11 and figure s8 in supplementary material).

#### Exploratory Symptom Analysis.

A significant group × symptom interaction (*P* = .04) was seen for positive symptoms when regressed against the extracted value from the left amygdala (−18 −10 −14). The ASD group showed a significant negative relationship between positive symptom score and activation change during the social compared to the gender condition (*r* = −50, *P* = .01) which was similar to the relationship in the CM group but different from the positive relationship in the SPD group (supplementary figure s9a). A significant group × symptom interaction (*P* = .01) was also seen for negative symptoms and the extracted value from the frontal cluster identified in the ASD < CM contrast (−18 −19 49). For this cluster the CM group showed a significant positive relationship with negative symptoms (*r* = .76, *P* = .02) while the SPD group showed a trend towards a significant negative relationship (*r* = −.43, *P* = .06; supplementary figure s9b).

#### Analysis of Gender vs Baseline Condition.

Analyses of the extracted gender vs baseline eigenvariates showed significantly increased activation in the ASD group in the left amygdala (−18 −10 −14) compared to the SPD and control groups (*P* = .003 and .01, respectively) and the left postcentral gyrus cluster (−18 −19 49) compared to the CM and control groups (*P* = .02 and *P* = .004, respectively). There were no instances of the SPD or CM groups showing greater activation than the other groups in the gender vs baseline analysis.

## Discussion

To our knowledge this is the first study directly comparing ASD and SPD using fMRI. The clinical groups all showed similar patterns of impairment compared to controls in negative symptoms and the social cognition tests, but clear differences were seen between the ASD and SPD groups using fMRI during the social judgment task. Differences between the ASD and SPD groups were also seen in the relationship between amygdala activation and positive symptoms. Our findings demonstrate that apparently similar clinical and neuropsychological features may be associated with quite distinct underlying brain mechanisms.

Although this is the first fMRI study comparing ASD and SPD, our findings are consistent with the 3 previous imaging studies which compared ASD and schizophrenia. Pinkham et al^42^ reported greater activation in right amygdala and left ventrolateral prefrontal cortex in non-paranoid individuals with schizophrenia compared to people with ASD during a trustworthiness judgment. In addition, a meta-analysis combining various mentalizing tasks showed greater activation in people with schizophrenia compared to those with ASD, albeit in different brain regions than we identified.^56^ Pinkham et al also reported qualitatively different factors underlying paranoia in ASD and schizophrenia,^57^ consistent with the opposing correlations between amygdala activation and positive symptoms that we report. This is also in keeping with a study showing that psychosis in autism was associated with different structural brain changes than psychosis alone.^58^

Recently, Ciaramidaro et al^41^ identified opposing patterns of brain activation in ASD and schizophrenia during intentionality assessment. Specifically, using stimuli which didn’t require the assessment of intention they identified hyperactivation in schizophrenia compared to controls in VMPFC and left posterior superior temporal sulcus. In contrast, using stimuli requiring an assessment of intention they found hypoactivation in the right posterior superior temporal sulcus in ASD. Similarly, Eack et al also identified increased ventromedial prefrontal and temporo-parietal junction activity in patients with schizophrenia compared to those with ASD during a visual perspective taking task.^43^ These findings are comparable to ours in that we also found opposing patterns of activation between groups in left temporoparietal regions and in the VMPFC, although the latter was only apparent in unmedicated individuals. However, Ciaramidaro et al’s findings also differ from ours in that they identified hyperactivation to a non-intentional stimulus in the schizophrenia group, whereas our findings are limited to explicit social judgments (ie, hyperactivation in the SPD group was not seen in the gender vs baseline analysis). This disparity between studies could relate to task differences, or to the difference between schizophrenia and SPD. It is possible that in people with SPD, this hyperactivation is limited to explicit social judgments, as opposed to also being inappropriately present during nonsocial judgments in schizophrenia.^41,59^ This may represent the mechanism by which individuals with SPD are spared some of the more severe symptomatology associated with schizophrenia.

We found hyperactivation in SPD compared to ASD in 2 regions we previously found to be activated in controls using the same task: the amygdala and the cerebellum. The amygdala has a range of functions in socio-emotional processing which include the detection of threat,^60^ so the increase in activation may represent an exaggeration of this response in SPD; with a relatively reduced response to such stimuli in the ASD group. However, we have previously found that the amygdala is activated by both affective and non-affective judgments, suggesting that the hyperactivation observed here may relate to a broader role of the amygdala in inferring the traits of others.^46^ Consistent with this, a recent meta-analysis found that activations in posterior cerebellum, which overlap strongly with those identified here, are also associated with tasks requiring participants to draw inferences about traits of others.^61^

We also identified increased activation in participants with SPD compared to those with ASD in the fusiform gyrus, a region strongly associated with face processing.^62^ On the left side we also identified a cluster in the intraparietal sulcus extending through the temporal gyri, including the superior temporal sulcus. The intraparietal sulcus and the superior temporal sulcus are known to be involved in assessing the intent of others,^63,64^ although more usually in the context of biological motion perception. Interestingly, increased activity in these regions has been reported in people with schizophrenia compared to controls when making judgments of a nonsocial, but not a social, nature^59^ and was also identified as hyperactive in schizophrenia compared to ASD by both Ciaramidaro et al^41^ and Eack et al.^43^

Although we did not identify clear group differences between either the ASD or SPD groups and the controls, results in the controls tended to lie between the 2 clinical groups, as did the findings for the CM group (figures 2 and 3). Given this, and the above, we suggest that our findings are consistent with the hypo- and hyper-mentalizing theory of ASD and schizophrenia.^36–38^ Further evidence for distinct patterns of pathophysiology comes from our finding that increased activation in the left amygdala is associated with increased positive symptoms in SPD, whereas the reverse is true in ASD. These opposite patterns of correlation are consistent with the hypo- and hyper-mentalizing theory of the autism and schizophrenia spectrums with the SPD group developing psychotic symptoms due to over-activation of amygdala, whereas the ASD group develops such symptoms due to under-activation of this region. It should be noted however that we made no attempt to correct for multiple comparisons for these exploratory analyses and therefore further research is required to confirm the differential symptom-function relationships which we report. At present, however, our results are in keeping with the idea that the schizophrenia and autism spectrums represent diametrical disorders of brain development, at least in regard to social cognition.^36,39,40^ Future studies investigating brain activation during other aspects of brain function known to be impaired in both conditions are required to determine if similar patterns are seen for other cognitive domains.

Irrespective of the exact nature of the underlying process, the differences we report carry important implications for clinical practice and classification. In particular it is important to note that clinical phenotypes can appear similar but arise from very different mechanisms and may therefore require quite different treatment approaches. This raises the prospect of developing treatments targeted at mentalizing styles, as opposed to clinical symptoms, an idea in keeping with the RDoC proposals.^65^ These findings also highlight the importance of considering SPD as a differential diagnosis for ASD and vice versa; it is therefore important that diagnostic services where these conditions may be met, especially those working with adults, contain access to skilled professional assessment of both sets of disorders.

We also identified people who met criteria for both ASD and SPD. This is consistent with previous work which reported that 23% of people with ASD met criteria for SPD.^32^ These “comorbid” individuals were more symptomatic than those with either condition alone, highlighting the importance of their identification. Interestingly, the fMRI findings for the CM group showed differences compared to the ASD group suggesting that they do not simply suffer from severe ASD. In contrast, there were no significant differences between the CM and SPD groups, which may indicate that they have a form of SPD. However, the numbers in this group are small making it difficult to draw firm conclusions. It is also possible that the definition of the CM group is reflective of the diagnostic tools that we employed and that more detailed clinical investigation could allocate members of this group more confidently into either one category or the other.

A number of limitations of the current study merit mention. The sample size is relatively small, especially the CM group, and a larger population may have identified more subtle differences. IQ differences were apparent between the groups, although the lack of correlation between IQ and the fMRI results suggests that this did not confound the results. In addition, ASD diagnoses were based upon DSM-IV criteria, and confirmed using the ADOS; we would ideally also have included a standardized developmental history but this was not practicable in this adult sample. In terms of the image analysis, the choice of threshold for our fMRI may be considered to be quite lenient raising the risk of type I error; however, we note that some differences between the groups were still apparent using the more stringent^55^ voxel based inference. Finally, it is likely that the gender judgment condition, although intended to remove non-affective face processing related activations, also contained an element of implicit social judgments, which may have reduced the differences between our groups when compared to the explicit judgment of approachability. The addition of a gender judgment using neutral stimuli with no affective content would perhaps have revealed greater differences between the groups.

Notwithstanding these limitations, we report marked overlaps between ASD and SPD in negative symptoms and social cognitive difficulties, but significant differences on examination of social brain activity using fMRI, consistent with the idea that these superficially similar conditions are associated with distinct underlying mechanisms.

## Supplementary Material

Supplementary data are available at *Schizophrenia Bulletin* online.

## Funding

This work was supported by a fellowship awarded to A.C.S. from the Wellcome Trust (WT802131MF) and by a research grant from Medical Research Scotland (206FRG). Further support came from the Shirley Foundation and the Dr Mortimer and Theresa Sackler Foundation.

## Supplementary Material

Supplementary_Material_resubmission
