# Supplementary material for: Dissociation of Brain Activation in Autism and Schizotypal Personality Disorder During Social Judgments
Source: Schizophr Bull. 2017 Jun 21;43(6):1220–8. doi: 10.1093/schbul/sbx083 (PMC5737648; doi:10.1093/schbul/sbx083)
Supplement: Supplementary_Material_resubmission [file sbx083_suppl_supplementary_material_resubmission.doc]

**Supplementary Material**

**Image Acquisition and Preprocessing**

All participants were scanned on a 1.5T GE Medical Systems Signa Scanner (GE Medical, USA). Axial, gradient-echo planar images (EPI) were acquired with repetition time (TR) of 2.5s, echo time (TE) of 40ms, matrix 64 x 64, field of view (FOV) of 240mm x 240mm and flip angle of 90 degrees. Thirty contiguous 5mm slices were acquired in an interleaved fashion within each TR. A T1 structural image was obtained using an MPRAGE sequence: 180 contiguous 1.2mm thick coronal slices were obtained in an interleaved fashion (TR 9.7ms, TE 4.0ms, matrix 192 x 192, FOV 240mm x 240mm, flip angle 8 degrees).

Image data were converted to NIfTI format and preprocessed using Statistical Parametric Mapping 8 software (SPM8 – www.fil.ion.ucl.ac.uk/spm/) running in MATLAB 2011b (The MathWorks, Inc). In each task the first four volumes of each run were discarded to avoid T1 equilibrium effects. The images were realigned to the mean EPI image and co-registered to the T1 structural image for each participant. The T1 and the functional images were then normalised to the standard Montreal Neurological Institute (MNI) template with a voxel size of 3mm x 3mm x 3mm and the functional images smoothed using an 8mm full width at half maximum Gaussian kernel.

**Summary of Social Cognition Results**

|  | ***ASD(1)*** | ***SPD(2)*** | ***CM(3)*** | ***Controls(4)*** | ***Between group differences*** |
| --- | --- | --- | --- | --- | --- |
| **Ekman 60**  *Anger*  *Disgust*  *Fear*  *Happiness*  *Sadness*  *Surprise* | 8 (1.5)  8 (4)  6.5 (4)  10 (0)  7 (3)  8.5 (3) | 8 (2)  8 (2.5)  6 (3.5)  10 (0)  8 (2)  9 (3) | 8 (3)  8 (2)  7 (4)  10 (0)  8 (2)  9 (1) | 9 (1)  8 (2)  8 (2)  10 (0)  8 (2)  9 (2) | 1<4*  1,2,3<4** |
| **Social judgements**  *Age*  *Approachability*  *Attractiveness*  *Distinctiveness*  *Intelligence*  *Trustworthiness* | 31 (1.5)  24.5 (8.5)  26.5 (6.5)  23 (3.5)  26 (4)  25.5 (4) | 31 (2)  26 (8.5)  26 (5)  22.5 (6.5)  27 (6.5)  23.5 (7) | 30 (2)  27 (9)  28 (3)  21 (5)  28 (3)  24 (5) | 31 (1)  29 (5)  29 (3)  25.5 (3.5)  28 (3)  25.5 (4) | 3<4**  1,2<4**  1,2<4**  1,2,3<4**  1,2<4** |

**Table s1:** Median (IQR) scores for each group for Ekman 60 and social judgement tasks

*p<0.01; **p<0.05

**Participant Characteristics for Imaging Component**

|  | ***ASD*** | ***SPD*** | ***CM*** | ***Controls*** |
| --- | --- | --- | --- | --- |
| *N* | 24 | 20 | 9 | 32 |
| *M:F* | 19:5 | 14:6 | 6:3 | 22:10 |
| *Age* | 40.5 (11.9) | 37.3 (9.4) | 35.8 (10.0) | 36.6 (9.5) |
| *Handedness* | 23:1 | 18:2 | 7:2 | 30:2 |
| *Yrs. education* | 16.4 (1.6) | 15.4 (2.0) | 16.1 (2.4) | 16.4 (2.0) |
| *Full-scale IQ** | 113.9 (17.1) | 106.4 (10.7) | 102.4 (23.6) | 117.9 (10.0) |
| *Antipsychotic use (yes:no)** | 2:22 | 5:15 | 3:6 | 0:32 |
| *CPZ equivalents** | 0 (50) | 0 (25-200) | 0 (25-400) | 0(0) |

**Table s2:** Characteristics of participants for fMRI study.

*differed significantly between the groups

**Participant performance during fMRI approachability task**

The mean scores for in-scanner performance for each group were: ASD=27.8, SPD=27.3, CM=29.4 and controls=30.7. Although these differences were not significant overall (F=1.9, p=0.13) there was some evidence that the ASD and SPD groups were more impaired than controls (p=0.06 and p=0.04 respectively).

**fMRI analysis: main effect of condition**

The main effect of condition shows the activation for the social versus gender contrasts when all four groups are considered together.

Significantly greater activation was found during the social > gender contrast bilaterally in the inferior frontal gyrus, superior medial prefrontal gyrus, insula, temporal poles, occipital regions and posterior cerebellum as well as in the left temporoparietal junction and right amygdala (Table s3 and Figure s1). No significant clusters were seen for the reverse contrast.

| **Locations of cluster peaks** | **MNI of peak** | | | **Extent** | **P_FWE_** | **Z_peak_** |
| --- | --- | --- | --- | --- | --- | --- |
| *Social > Gender* |  |  |  |  |  |  |
| L inferior frontal gyrus  - p orbitalis and p triangularis | -54 | 32 | -2 | 218 | 0.01 | 3.99 |
| R. inferior frontal gyrus  - p triangularis | 51 | 29 | -5 | 237 | 0.01 | 4.57 |
| L. & R. superior medial gyrus | -9 | -32 | 58 | 1121 | <0.001 | 5.88 |
| L. ant. inferior temporal gyrus | -48 | 2 | -35 | 146 | 0.048 | 5.11 |
| L. post. middle temporal gyrus | -57 | -43 | 1 | 346 | 0.002 | 4.43 |
| L. & R. calcarine gyrus | -9 | -85 | 1 | 1953 | <0.001 | ∞ |
| L. cerebellum | -39 | -67 | -44 | 245 | 0.009 | 5.74 |
| R. amygdala  *Gender > Social* | 15 | -4 | 14 |  | 0.01 ^SVC^ | 3.52 |
| No significant clusters |  |  |  |  |  |  |

**Table s3:** Brain activations during social versus gender contrast across all groups

Significance values reported are cluster values FWE-corrected for whole brain volume unless indicated using ^SVC^ when significance is reported at voxel level FWE corrected for amygdala volume; L. = left; R. = right; ant. = anterior; post. = posterior


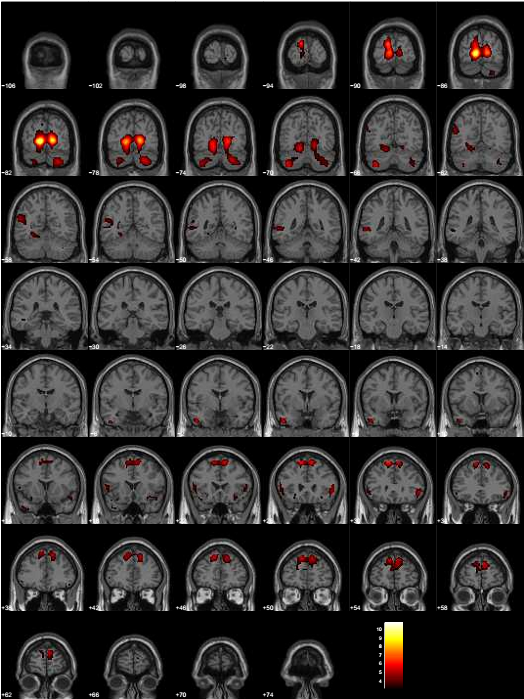


**Figure s1:** Clusters of activation for social > gender contrast across all four groups

No significant clusters were seen for gender > social

#### fMRI analysis: Within group activations

*Within Controls*

| **Locations of cluster peaks** | **MNI of peak** | | | **Extent** | **P_FWE_** | **Z_peak_** |
| --- | --- | --- | --- | --- | --- | --- |
| *Social > Gender* |  |  |  |  |  |  |
| L. & R. superior medial gyrus | 9 | 32 | 58 | 183 | 0.02 | 4.19 |
| L. & R. superior medial gyrus | 12 | 56 | 34 | 202 | 0.02 | 3.76 |
| L. & R. lingual gyrus | 12 | -82 | 1 | 472 | <0.001 | 5.86 |
| L. cerebellum  - VIIa Crus II | -36 | -70 | -44 | 201 | 0.02 | 4.24 |
| R. cerebellum  - VIIa Crus II | 30 | -82 | -44 | 114 | 0.09 | 3.97 |
| *Gender > Social* |  |  |  |  |  |  |
| No significant clusters |  |  |  |  |  |  |

**Table s4:** Brain activations during social versus gender contrast for control group


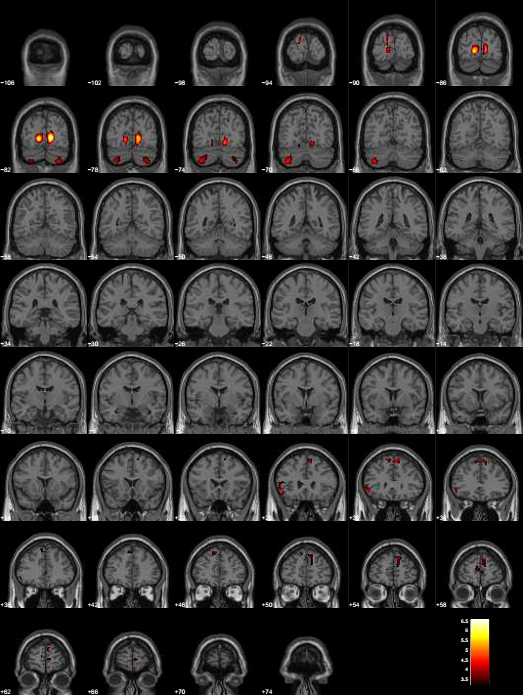


**Figure s2:** Activations within control group for social > gender contrast

No significant clusters were seen for gender > social

*Within ASD group*

| **Locations of cluster peaks** | **MNI** | | | **Extent** | **P_FWE_** | **Z_peak_** |
| --- | --- | --- | --- | --- | --- | --- |
| *Social > Gender* |  |  |  |  |  |  |
| L. calcarine gyrus | -9 | -85 | 4 | 118 | 0.08 | 5.77 |
| *Gender > Social* |  |  |  |  |  |  |
| No significant clusters |  |  |  |  |  |  |

**Table s5:** Brain activations during social versus gender contrast for ASD group


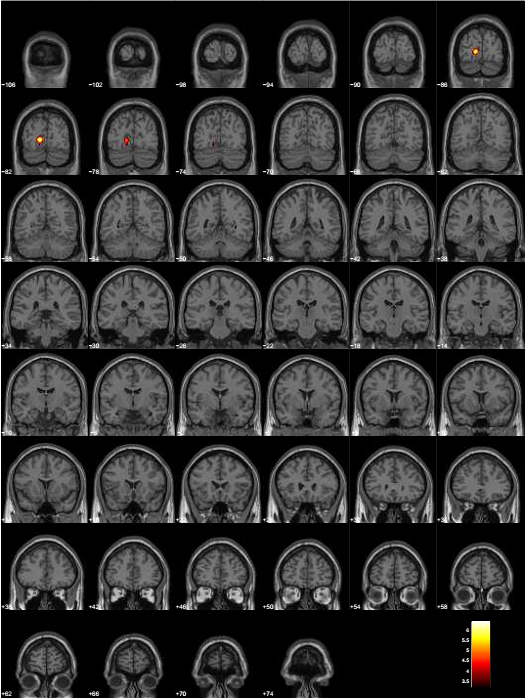


**Figure s3:** Activations within ASD group for social > gender contrast

No significant clusters were seen for gender > social

*Within SPD group*

| **Locations of cluster peaks** | **MNI of peak** | | | **Extent** | **P_FWE_** | **Z_peak_** |
| --- | --- | --- | --- | --- | --- | --- |
| *Social > Gender* |  |  |  |  |  |  |
| L. superior frontal gyrus | -12 | 32 | 58 | 233 | 0.01 | 4.48 |
| L. & R. superior medial gyrus | -6 | 56 | 34 | 155 | 0.04 | 4.33 |
| L. inferior frontal gyrus  - p. orbitalis and p. triangularis | -54 | 17 | 4 | 200 | 0.02 | 4.6 |
| R. inferior frontal gyrus  - p. triangularis | 51 | 35 | 25 | 232 | 0.01 | 5.52 |
| L. inferior temporal gyrus | -48 | -1 | -38 | 135 | 0.06 | 4.61 |
| L. middle temporal gyrus | -63 | -40 | 1 | 110 | 0.096 | 4.68 |
| L. & R. calcarine gyrus | -9 | -85 | 1 | 2422 | <0.001 | 5.62 |
| L. caudate & pallidum | -15 | 5 | -5 | 136 | 0.058 | 3.86 |
| L. hippocampus | -3 | -25 | -26 | 139 | 0.054 | 4.49 |
| R. amygdala | 15 | -4 | -14 |  | 0.02^SVC^ | 3.38 |
| *Gender > Social* |  |  |  |  |  |  |
| No significant clusters |  |  |  |  |  |  |

**Table s6:** Brain activations during social versus gender contrast for SPD group


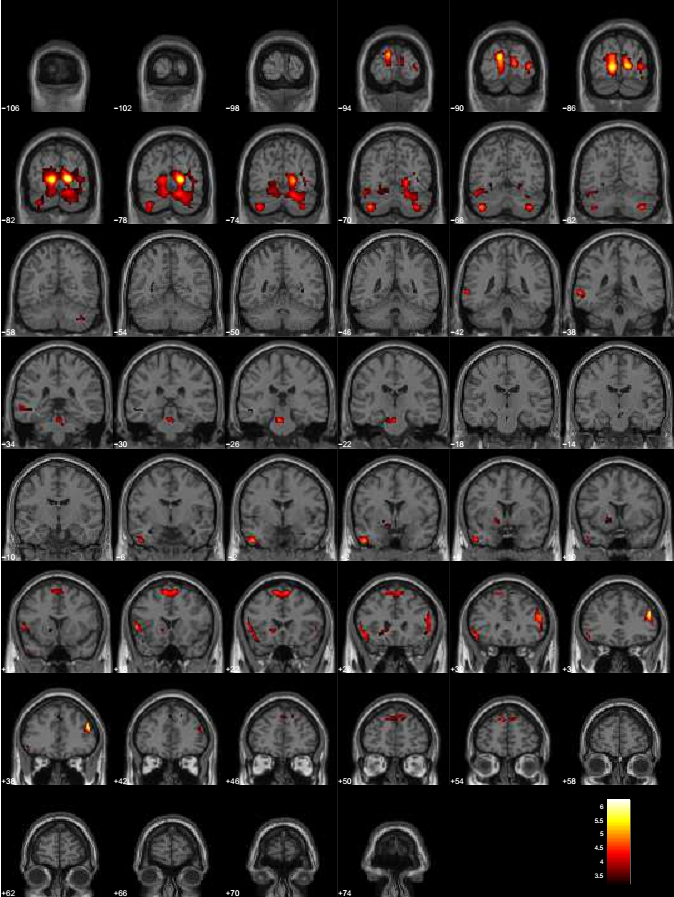


**Figure s4:** Activations within SPD group for social > gender contrast

No significant clusters were seen for gender > social


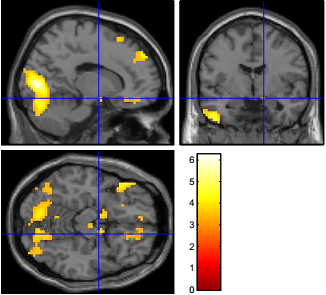


**Figure s5:** Location of right amygdala activation (MNI: 15 -4 -14) within SPD group for social > gender contrast

*Within CM group*

No significant regions of activation were seen in the CM group in either the social > gender or the gender > social contrast.

**fMRI analysis: Group x condition interaction between ASD and control groups**

| **Locations of cluster peaks** | **MNI of peak** | | | **Extent** | **P_FWE_** | **Z_peak_** |
| --- | --- | --- | --- | --- | --- | --- |
| *ASD > Control* |  |  |  |  |  |  |
| No significant clusters |  |  |  |  |  |  |
| *ASD < Control* |  |  |  |  |  |  |
| R. cerebellum  - VI, VIIa Crus I and II | 30 | -58 | -44 | 126 | 0.050 | 4.16 |
| L. cerebellum  - VI, VIIa Crus I and II | -45 | -55 | -41 | 329 | 0.07 | 3.52 |

**Table s7:** Brain regions showing differences in the relative increase in activation seen using the social > gender contrast between the ASD and control groups


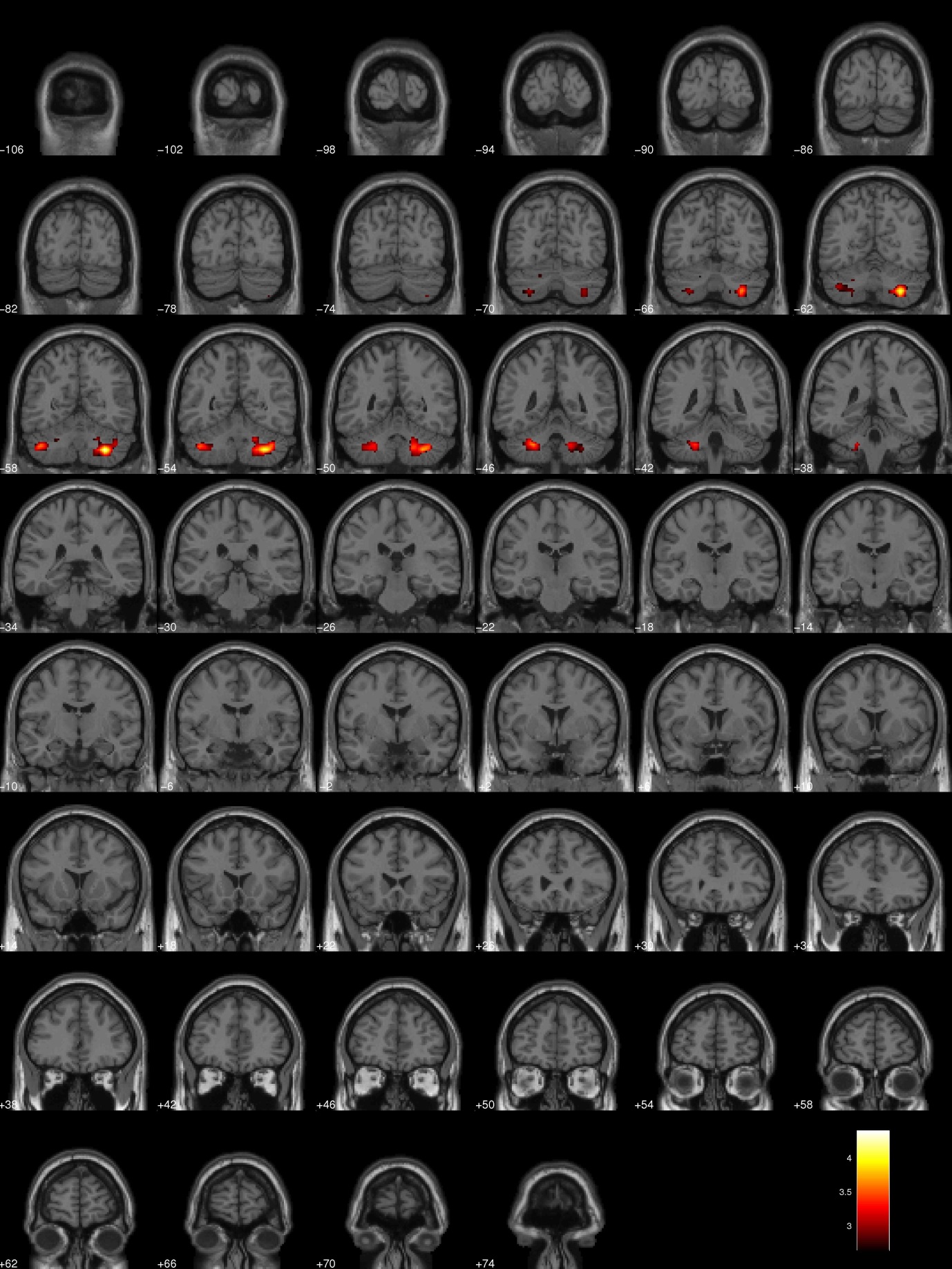


**Figure s6:** Clusters showing less increase in activation in the ASD group compared to controls in the social > gender contrast.

**fMRI analysis: Group x condition interaction between ASD and SPD groups**

| **Location of cluster peaks** | **MNI of peak** | | | **Extent** | **P_FWE_** | **Z_peak_** |
| --- | --- | --- | --- | --- | --- | --- |
| *ASD > SPD* |  |  |  |  |  |  |
| No significant clusters |  |  |  |  |  |  |
| *ASD < SPD* |  |  |  |  |  |  |
| L. intraparietal sulcus | -24 | -52 | 31 | 403 | 0.04 | 3.55 |
| L. cerebellum  - anterior, VI, VIIa Crus I&II, VIIb | -15 | -40 | -38 | 652 | 0.005 | 4.18 |
| R. cerebellum  - VI, VIIa Crus I, VIIIb | 33 | -64 | -44 | 1554 | <0.001 | 4.54 |
| L. amygdala | -18 | -10 | -14 |  | 0.046^SVC^ | 3.00 |

**Table s8:** Brain regions showing differences in the relative increase in activation seen using the social > gender contrast between the ASD and SPD groups.


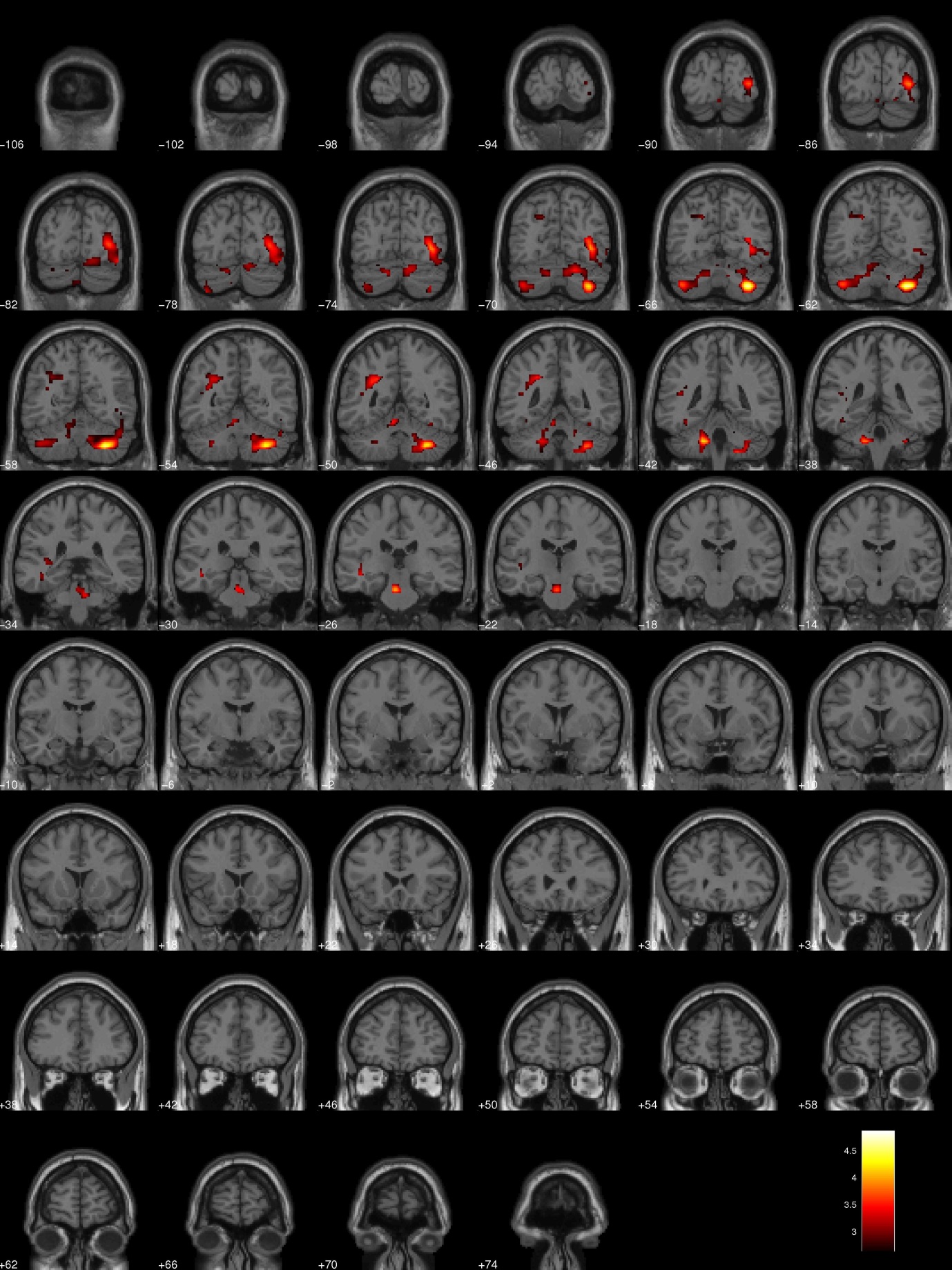


**Figure s7:** Clusters showing greater increase in activation in the SPD group compared to the ASD group using the social > gender contrast

**fMRI analysis: Group x condition interaction between ASD and CM groups**

| **Locations of cluster peaks** | **MNI of peak** | | | **Extent** | **P_FWE_** | **Z_peak_** |
| --- | --- | --- | --- | --- | --- | --- |
| *ASD > CM* |  |  |  |  |  |  |
| No significant clusters |  |  |  |  |  |  |
| *ASD < CM* |  |  |  |  |  |  |
| L. postcentral gyrus | -18 | -19 | 49 | 844 | 0.001 | 3.94 |
| R. cerebellum (VI, VIIa, VIIb) | 24 | -55 | -41 | 404 | 0.04 | 3.84 |

**Table s9:** Brain regions showing differences in the relative increase in activation seen using the social > gender contrast between the CM and the ASD groups.

**fMRI analyses incorporating only participants naïve to antipsychotic medication**

|  | ***ASD*** | ***SPD*** | ***CM*** | ***Controls*** |
| --- | --- | --- | --- | --- |
| *N* | 22 | 15 | 6 | 32 |
| *M:F* | 18:4 | 9:6 | 5:1 | 22:10 |
| *Age* | 41.2 (11.9) | 37.5 (8.9) | 35.9 (10.7) | 36.6 (9.5) |
| *Handedness* | 21:1 | 13:2 | 5:1 | 30:2 |
| *Yrs. education* | 16.5 (1.5) | 15.8 (1.9) | 16.5 (2.3) | 16.4 (2.0) |
| *Full-scale IQ* | 115.0 (17.1) | 103.5 (10.7) | 102.5 (23.6) | 117.9 (10.0) |

**Table s10:** Participant characteristics for individuals who were antipsychotic naïve

For the ASD versus SPD analysis, the cluster in the right cerebellum remained significant and stretched across the midline into the left cerebellum, and a new significant cluster in the ventromedial prefrontal cortex (VMPFC) extending into the left putamen was found to be significantly more activated in the SPD group than the ASD group when making social as compared to gender judgements (Table s11 and Figure s8)

| **Locations of cluster peaks** | **MNI of peak** | | | **Extent** | **P_FWE_** | **Z_peak_** |
| --- | --- | --- | --- | --- | --- | --- |
| *ASD > SPD* |  |  |  |  |  |  |
| No significant clusters |  |  |  |  |  |  |
| *ASD < SPD* |  |  |  |  |  |  |
| R & L superior orbital gyri | 18 | 35 | -17 | 470 | 0.02 | 3.80 |
| R. cerebellum  - VI, VIIa Crus I, VIIIb  L. cerebellum  - VI, VIIa Crus I and II | 33 | -64 | -44 | 438 | 0.03 | 3.87 |

**Table s11:** Brain regions showing differences in the relative increase in activation seen using the social > gender contrast between the ASD and SPD groups when only antipsychotic naïve participants included


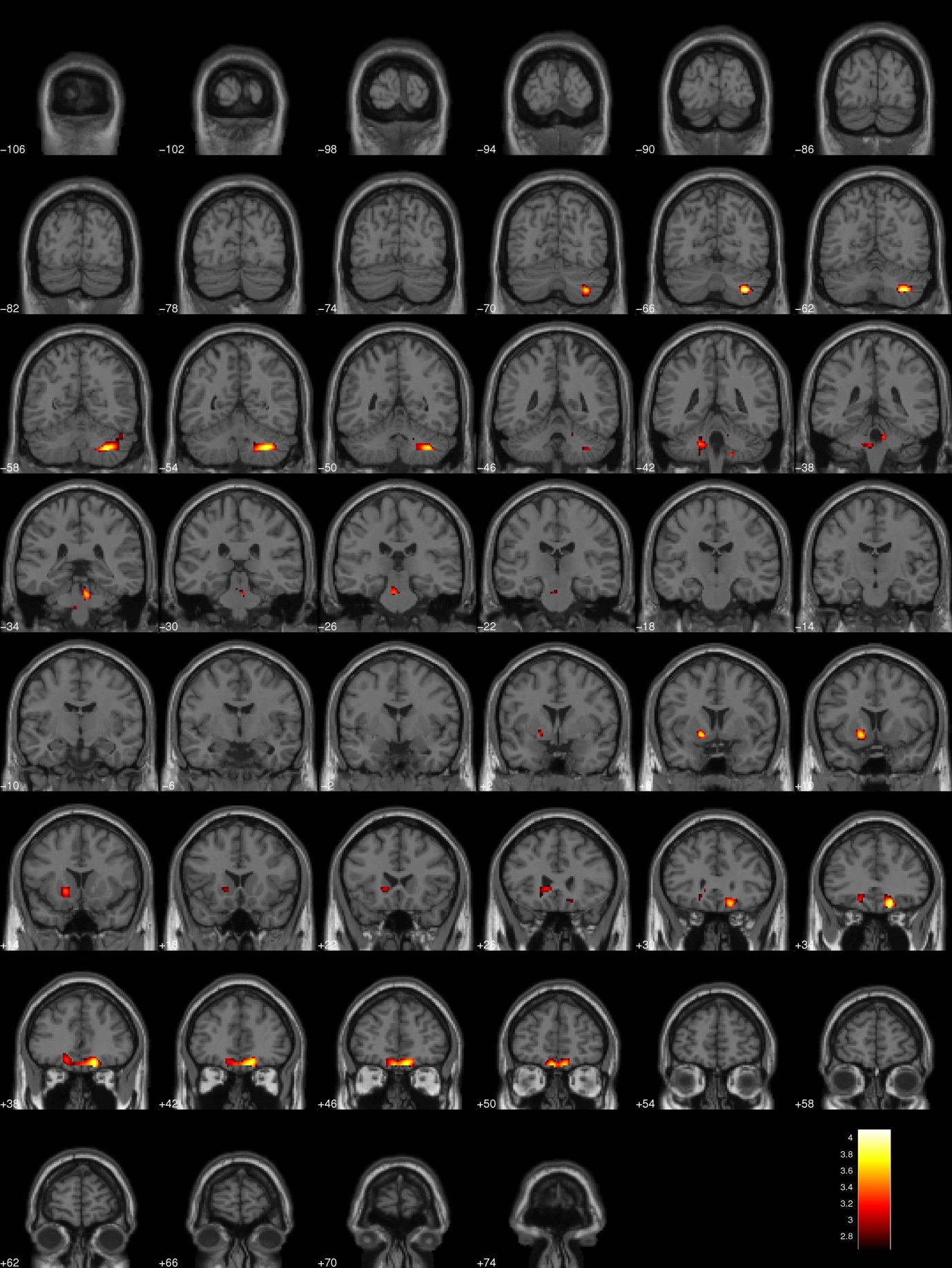


**Figure s8:** Clusters showing greater increase in activation in the SPD group compared to the ASD group using the social > gender contrast in antipsychotic naïve participants only

**Exploratory Symptom Analysis**

ab

**Figure s9:** Relationship between (a) left amygdala activation and positive symptoms; and (b) left postcentral gyrus activation and negative symptoms
